# New Appliances & Things Medical

**Published:** 1909-07-10

**Authors:** 


### NEW APPLIANCES & THINGS MEDICAL.

### CAMPBELL'S SOUPS.

We have received from the manufacturers, the Joseph Campbell Company, of Camden, New Jersey, U.S.A., and 6 and 8 Bouverie Street, London, E.C., samples of their condensed soups, which we have now investigated and can recommend to medical practitioners and others responsible for invalid dietaries. Of the twenty-one varieties of these soups now upon the market, we have received and examined five which are considered most appropriate to hospitals and the sick-room. These are styled respectively "Clam Chowder," "Clam Bouillon," "Oxtail," "Mock Turtle," and "Mutton Broth." We have found them easy to prepare, pleasant to taste, and very digestible, and we can well believe the statements made by the manufacturers as to the purity of the ingredients and the cleanliness and security from contamination of the method of preparation. Chemical preservatives are rendered unnecessary by the submission of the sealed vessels of soup to a temperature of 240° F. after their contents have been passed from the blending kettles into the tins through glass-lined pipes. The ease and rapidity with which these soups can be prepared, and their flavour and digestibility, render them suitable for invalids as well as for ordinary household use. We have further received from the same makers sample bottles of their tomato ketchup and spiced mustard, which have also impressed us favourably.

### SIXTEENTH INTERNATIONAL CONGRESS OF MEDICINE—BUDAPEST.

August 29th—September 4th, 1909.

The travelling arrangements to and from the Budapest Congress have now been issued. The *Cartes d'identité* enabling members to avail themselves of the reductions have been issued to everyone who has paid his subscription. The South-Eastern and Chatham Railway Company quote specially reduced rates for parties of not less than twenty passengers travelling together on the outward journey to the Congress, but makes no reduction to members travelling individually. The Zeeland Steamship Company, which runs through carriages from Flushing to Vienna in connection with the day and night services from Victoria viâ Queenboro', will also allow a reduction on the ordinary fare for parties who travel together. The Great Eastern Company issues circular tour tickets as follows : London, Harwich, Hook of Holland, Rotterdam, Cologne, Mayence, Frankfort o/M, Passau, Linz, Vienna, Budapest, Vienna, Dresden, Hanover, Salzbergen, Hook of Holland, Harwich, and London. The cost of these tickets is £10 14s. 5d., and they are available for ninety days, second-class rail in England, first-class on the G. E. Co.'s steamers, second-class rail on the Continent and first-class on the Rhine steamers, or second-class rail between Cologne and Mayence. By the night service leaving Liverpool Street at 8.30 p.m. there is a through carriage with a restaurant car from the Hook of Holland to Frankfort (5.48 a.m. to 4.10 p.m.) and a sleeping car from Frankfort to Vienna (4.37 p.m. to 7.30 a.m.). If the journey is taken viâ Dresden there is a through carriage with restaurant car from the Hook of Holland to Hanover (5.25 a.m. to 3.5 p.m.), a through carriage with restaurant car from Hanover to Leipsic (3.5 p.m. to 10.2 p.m.) and a sleeping car from Dresden to Vienna (10.2 p.m. to 7.40 p.m.). The G.E.R. allow a further reduction of fare when twenty members of a party travel together. Mr. J. Y. W. Macalister issues a circular from the Royal Society of Medicine stating that he has organised a party for the Fellows of the Royal Society of Medicine and for the friends of Fellows, although they are not connected with the Society, who are properly vouched for. The party will be accompanied by an experienced courier. It will travel by the train de luxe both on the outward and homeward journeys, and will be lodged in the best hotels. The inclusive charge is thirty-six guineas viâ Ostend and thirty-eight guineas viâ Calais. The official reductions are fifty per cent. on the French lines, if application be made at least three days before the ticket is required upon the pink form issued. The form must be stamped at the Congress before it is available for the return journey. The Royal Hungarian State Railways make a reduction of fifty per cent. to members furnished with green card of identity. It appears from the instructions issued that very few members of the British Section will be able to obtain the authorised reductions without breaking their journey at the frontier towns, as it has been found impossible to establish any system of through tickets at reduced fares.

The Hospital for Invalid Gentlewomen, established at 90 Harley Street fifty-six years ago by Viscountess Canning and Miss Florence Nightingale, is closed, owing to the expiration of the lease. A new hospital for thirty-two patients is in course of construction at 19 Lisson Grove, and will be opened for the reception of patients towards the end of the year. Five thousand pounds are still needed to complete the building and equipment. Donations should be sent to W. C. Bridgeman, Esq., M.P., 13 Mansfield Street, W.

